# Surgical removal of part of an occluder to treat iatrogenic coarctation of the aorta: a case report

**DOI:** 10.1186/s12893-020-0682-6

**Published:** 2020-01-17

**Authors:** Zhongwei Sun, Dongxu Li, Yabo Wang, Qi An

**Affiliations:** Department of Cardiovascular Surgery, West China Hospital, Sichuan University, No. 37 Guo Xue Xiang, Chengdu, 610041 Sichuan China

**Keywords:** Patent ductus arteriosus, Iatrogenic, Coarctation of the aorta, Surgical retrieval, Case report

## Abstract

**Background:**

Iatrogenic aortic stenosis is a serious complication and potentially fatal due to erosion of the aortic wall. Timely management is necessary to prevent complications.

**Case presentation:**

A 2-year-old boy underwent surgery to remove part of an Amplatzer occluder after patent ductus arteriosus (PDA) device embolization in the thoracic aorta. He exhibited moderate to severe obstruction with erosion of the intimal layer of the aorta caused by the device, part of which was retrieved surgically with restructuring of the thoracic aorta segment and occluder remnant. The patient’s postoperative course was uneventful.

**Conclusions:**

When possible, retrieving only part of an embolized device can be advocated because it reduces the risk of aortic and pulmonary artery damage.

## Background

Transcatheter closure of patent ductus arteriosus (PDA) is an effective and less invasive method when compared to surgical intervention [1]. Moreover, percutaneous PDA closure during infancy is feasible and is associated with few major or catastrophic adverse events [2]. However, iatrogenic aortic stenosis is a serious complication and potentially fatal due to the erosion of the aortic wall, necessitating timely management. We report a unique method to treat iatrogenic coarctation of the aorta (CoA) after transcatheter closure of PDA, by cutting out part of the disk under deep hypothermic circulatory arrest and performing an end-to-end anastomosis.

## Case presentation

**Fig. 1:**
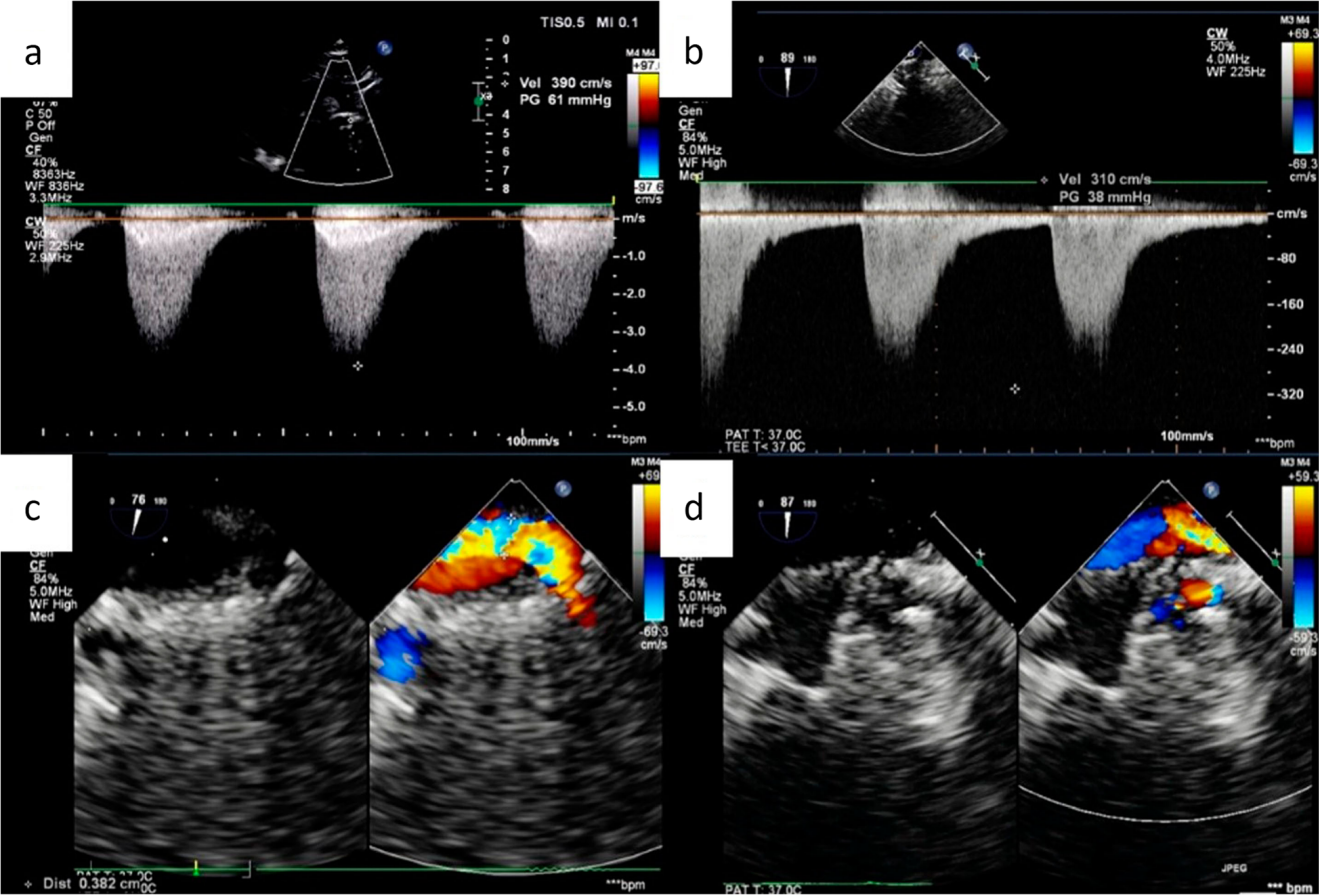
Preoperative transthoracic echocardiography showed the descending aorta with a pressure difference across the thoracic aorta of 61 mmHg (**a**) and these findings were confirmed by transesophageal echocardiography (**b** and **c**). The postoperative echocardiography exhibited abolition of the descending aortic flow rushing with no pressure difference (**d**)

At the age of 2 years, a thin and weak boy presented to the authors’ hospital with thoracic aortic flow obstruction. At about 4 months of age, he had received a transcatheter Amplatzer occluder at a local hospital to block the PDA. Over time, the occluder prominently bulged out of the descending aorta, causing blood flow obstruction as indicated by an echocardiogram conducted at the author’s hospital. Preoperative transthoracic echocardiography revealed iatrogenic CoA, with a forward blood flow speed of 390 cm/s, and a thoracic aorta pressure difference of 61 mmHg (Fig.1a). Transesophageal echocardiography (Additional file 1: Video S1) also showed similar results, revealing significant iatrogenic CoA with forward blood flow rushing at 310 cm/s and the narrow aorta diameter, with a width of 0.382 cm (Fig.1b, c). Because the embolization device caused severe aortic blood flow obstruction and the risk of aortic rupture, the patient eventually underwent surgery.

**Fig. 2:**
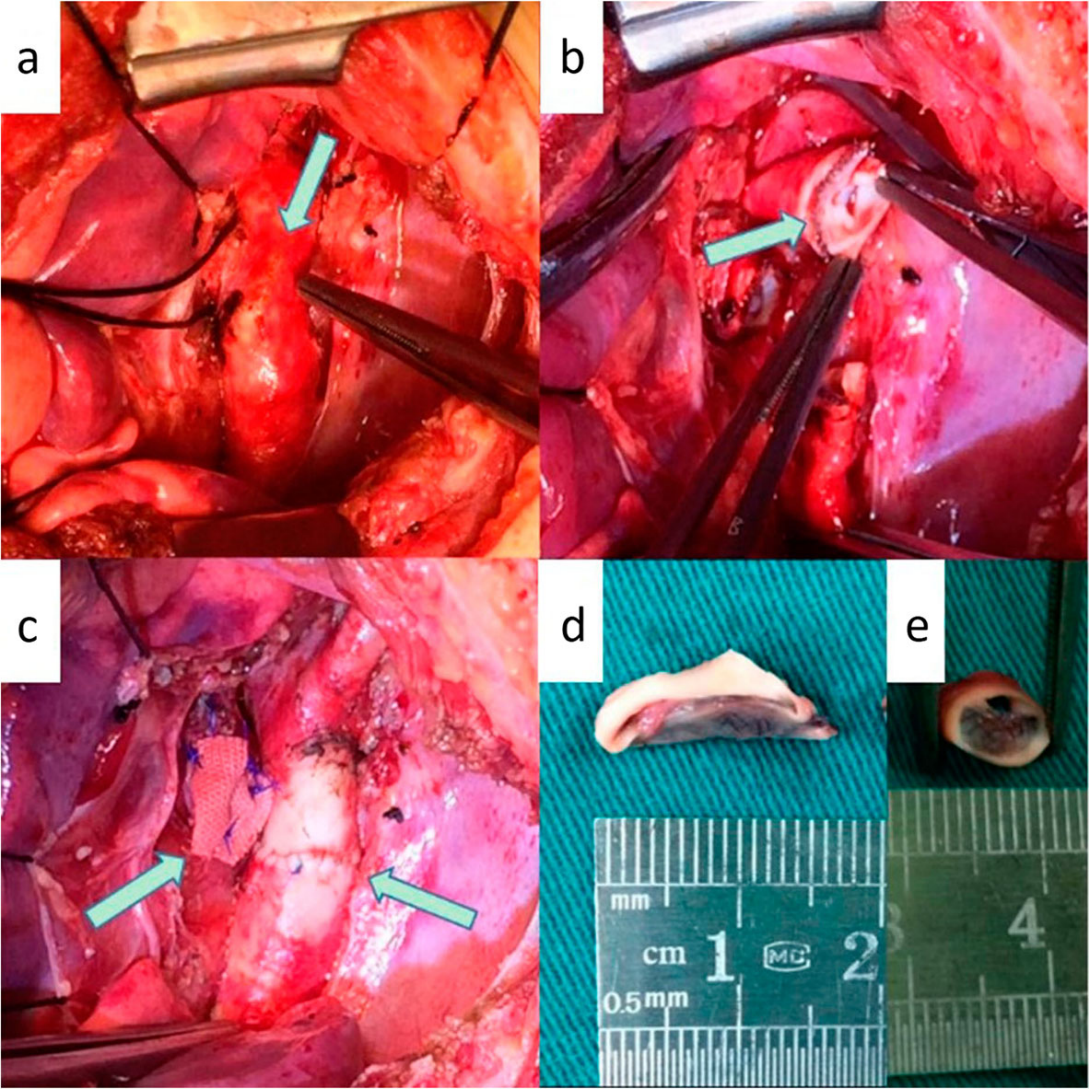
The embolized PDA device with the linear trace was visible palpable and the aortic wall in contact region was clearly thin and white (**a**). The device was found to be completely endothelialized (**b**). End-to-end anastomosis of the aorta was performed (**c**, right arrow) and the occluder stump was wrapped and sutured (**c**, left arrow). The disk edge and waist parts of occluder are clearly visible (**d**, **e**)

The patient was treated with left posterolateral intercostal incision. First, the surrounding tissue was separated carefully, making the ductus occluder causing embolization visible on the linear trace. The marks on the thoracic aorta could be clearly touched, and the aortic wall at the region of contact was thin and white (Fig. 2a). Second, a transverse aortotomy was created under deep hypothermic circulatory arrest, and the proximal and distal ends were occluded using vascular clamps. The occluder was completely covered by endothelial cells which grew into the wall of the thoracic aorta, making it difficult to separate. The contacts eroded by the disk could be seen clearly at the front (Fig. 2b). Importantly, if the damaged tissue is retained for continued use, aortic dissection and aneurysm may occur at any time due to the impact of intra-aortic blood flow, so we removed the damaged part and anastomosed the thoracic aorta at both ends (Fig. 2c, right arrow). The occluder was firmly bonded to the PDA and the left pulmonary artery and therefore, the disk was cut off at the aortic and waist parts of the occluder (Fig. 2d, e). The other parts of the waist and disk were retained in the left pulmonary artery. It was then wrapped and sutured using a polyester patch to prevent the occluder stump from falling off and abrading the aortic wall (Fig. 2c, left arrow). Improved blood flow in the thoracic aorta was clearly visible, and no pressure gradient was found with postoperative ultrasound review (Fig. 1d and Additional file 2: Video S2). Six months postoperatively, the patient was doing well and there were no differences in physical development as compared to healthy children of the same age, to the satisfaction of his parents.

## Discussion and conclusions

PDA is a pathological vascular structure in which the ductus arteriosus is not closed and remains open after birth. It exists as a blood shunt, from left to right, from proximal descending aorta to main pulmonary artery. It is important to have a full understanding of the pathophysiology, clinical implications, and management of PDA [3]. PDA with hemodynamic significance is worth discussing. It should take into account the gestational age and the risk of high or low organ perfusion [4]. If a such PDA is left untreated, in the most extreme cases pulmonary artery dissection aneurysms eventually develop into pericardial hemorrhage [5]. With the development of PDA, pulmonary hypertension may occur and develop further into Eisenmenger syndrome. Lung transplantation may be required during the Eisenmenger syndrome stage [6]. Therefore, PDA needs therapeutic intervention.

There were three possible ways to manage the patient described in this report. In addition to drug therapy, transcatheter closure and open surgery both have advantages and disadvantages. Regarding transcatheter closure, occluder embolism, aortic dissection, and occluder swelling to pulmonary artery to cause stenosis and even vascular blockage are possible complications [7, 8]. According to the Catheterization for Congenital Heart Disease Adjustment for Risk Method, adverse events such as embolization, stenosis or occlusion and even fatal hemopericardium were on the higher end of the severity level scale [9]. Transcatheter closure is often used after drug treatment failure, and it is used as an alternative to open surgery for premature infants.

Serious transcatheter closure-related complications, such as embolization and arterial injury [10], cannot be ignored. Embolism is commonly caused by insertion of an undersized device, incorrect positioning, and erroneous operation [11–13], whereas arterial injuries are caused by iatrogenic coarctation or aortic dissection due to smaller aortic dimension, poor arterial elasticity, and aortic wall abrasion secondary to the disk at the aortic side, among others [14, 15]. Therefore, a correct-sized occluder should be selected when performing transcatheter closure and the condition at post-implantation morphologically examined [8]. The use of a balloon-assisted release method intraoperatively has been recommended to prevent the occluder from protruding to the aorta during the PDA catheter occlusion [14]. Ireneusz et al. [16] reported an interesting case of a 10-month-old girl with severely narrowed aorta after the PDA transcatheter occlusion, requiring an emergency surgery to remove the occluder to prevent severe cardiac failure. Hypertensive arterial changes occurred over coarctation, and apoptosis was observed in other organs below the aorta. Unfortunately, the child eventually died of irreversible multiple organ damage. Therefore, iatrogenic coarctation of the aorta after a PDA occlusion should be detected and treated immediately. Postoperatively, if arterial damage persists, embolized devices may also cause erosion, even aortic dissection, and the tissue structure of the blood vessel wall in contact with the device becomes weak over time [17]. Attempting transcatheter removal may cause irreparable consequences such as rupture of thoracic or pulmonary arteries for cases that have developed fusion between the occluder and vessel. It is, therefore, suggested that in such instances invasive surgery should be the main choice. This case is similar to the case reported by R. R. Tripathi et al., but there are differences in treatment [17]. They chose to remove the occluder as a whole, and we preferred to remove it partially, and leave the rest in the body. If the occluder had been removed completely in our case, it would have caused pulmonary artery damage. This would have been difficult to repair and caused even further narrowing after repair. Therefore, surgical decisions for iatrogenic CoA and postoperative follow-up is the first priority. Although iatrogenic CoA and surgical protocols to retrieve the embolized PDA occluder have been described, to our knowledge this is the first report to depict a case in which only a part of an embolized device was retrieved while the remainder was sutured and wrapped. The polyester patch is a prophylactic implant designed to isolate the rough stump of the occluder and prevent damage to the adjacent aorta. It is preferred over the bovine patch because of the following reasons: First, the patch is wrapped outside the vessel and not in direct contact with blood. Thus, the characteristics of an ideal vascular prosthesis, such as puncture-free bleeding, need not be considered [18]. Second, calcification may occur in the bovine patch [19], and consequently its protective effect may disappear. Finally, the polyester patch is relatively more economical than the bovine patch.

In summary, aortic erosion after iatrogenic CoA due to PDA transcatheter closure is rare but nevertheless results in a high rate of aortic rupture. Transcatheter retrieval can lead to fatal consequences such as aortic rupture in some cases. In fact, surgical procedures involving left posterolateral thoracotomy can be considered for such a delicate situation. When possible, retrieving only part of an embolized device can be advocated, as it reduces the risks of aortic and pulmonary artery damage.

## Supplementary information

**Additional file 1: Video S1.** Preoperative transesophageal echocardiography showing an iatrogenic coarctation of the aorta with forward blood flow rushing.

**Additional file 2: Video S2.** Postoperative transesophageal echocardiography exhibiting abolition of the descending aortic flow rushing.

## Data Availability

The data supporting the findings of this study are available within the article.

## Abbreviations

CoA: Coarctation of the aorta
PDA: Patent ductus arteriosus
